# LncRNA KIFAP3-5:1 inhibits epithelial-mesenchymal transition of renal tubular cell through PRRX1 in diabetic nephropathy

**DOI:** 10.1007/s10565-024-09874-5

**Published:** 2024-06-13

**Authors:** Lei Du, Yinfei Lu, Jingyi Wang, Yijia Zheng, Huan Li, Yunfei Liu, Xiaoling Wu, Jieling Zhou, Lei Wang, Linlin He, Jiasen Shi, Liu Xu, Xizhi Li, Qian Lu, Xiaoxing Yin

**Affiliations:** Jiangsu Key Laboratory of New Drug Research and Clinical Pharmacy, Xuzhou Medical University, 209 Tongshan Road, Xuzhou, 221004 Jiangsu China

**Keywords:** KIFAP3-5:1, EMT, renal fibrosis, PRRX1, Diabetic nephropathy

## Abstract

Long noncoding RNAs play an important role in several pathogenic processes in diabetic nephropathy, but the relationship with epithelial-mesenchymal transition in DN is unclear. Herein, we found that KIFAP3-5:1 expression was significantly down-regulated in DN plasma samples, *db/db* mouse kidney tissues and high glucose treated renal tubular epithelial cells compared to normal healthy samples and untreated cells. Overexpression of KIFAP3-5:1 improved renal fibrosis in *db/db* mice and rescued epithelial-mesenchymal transition of high glucose cultured renal tubular epithelial cells. The silence of KIFAP3-5:1 will exacerbate the progression of EMT. Mechanistically, KIFAP3-5:1 was confirmed to directly target to the -488 to -609 element of the PRRX1 promoter and negatively modulate PRRX1 mRNA and protein expressions. Furthermore, rescue assays demonstrated that the knockdown of PRRX1 counteracted the KIFAP3-5:1 low expression-mediated effects on EMT in hRPTECs cultured under high glucose. The plasma KIFAP3-5:1 of DN patients is highly correlated with the severity of renal dysfunction and plays an important role in the prediction model of DN diseases. These findings suggested that KIFAP3-5:1 plays a critical role in regulation of renal EMT and fibrosis through suppress PRRX1, and highlight the clinical potential of KIFAP3-5:1 to assist in the diagnosis of diabetic nephropathy.

**Graphical abstract:**

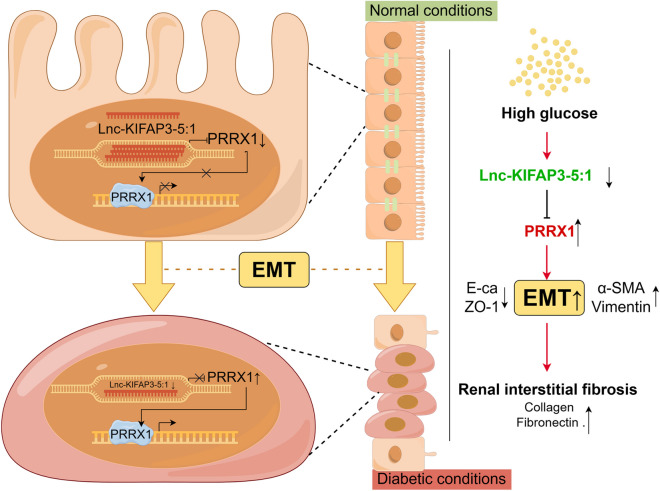

**Supplementary Information:**

The online version contains supplementary material available at 10.1007/s10565-024-09874-5.

## Introduction

Diabetic nephropathy (DN) represents a significant microvascular complication arising from diabetes mellitus (DM), serving as a primary contributor to the development of end-stage renal disease (ESRD) globally (Sanchez-Nino et al. 2020; Wang et al. 2021). Unfortunately, the exact pathogenesis of DN remains unclear. Renal interstitial fibrosis (RIF) serves as the predominant pathological underpinning for the advancement of DN towards ESRD. (Chevalier 2016). Epithelial-to-Mesenchymal Transition (EMT) is an essential initiator of renal interstitial fibrosis. This condition is characterized by the absence of epithelial surface markers such as ZO-1 and E-cadherin, a diminished capacity for renal tubular epithelial cells to adhere, and the upregulation of mesenchymal markers, including α-SMA and Vimentin (Xie et al. 2022). Prior research has established that the EMT of renal tubular epithelial cells serves as a primary contributor to RIF in DN (Bridoux et al. 2021). Therefore, inhibiting or reversing the occurrence of EMT can be of great significance in delaying the progression of DN.

Long non-coding RNAs (LncRNA) are RNA transcripts that exceed a length of 200 nucleotides. They play an important regulatory role in various physiological and pathological processes, including cell proliferation (Li et al. 2021; Zhang et al. 2021; Wu et al. 2023; Huang et al. 2022). LncRNAs, through their interactions with other RNA molecules or proteins, play a crucial role in the regulation of gene expression at both transcriptional and post-transcriptional levels (Xu et al. 2022; Han et al. 2022). Multiple studies have consistently shown a strong association between lncRNA and the etiology and advancement of DN. LncRNAs TUG1 and SOX2OT have been shown to have a renoprotective effect against DN (Chen et al. 2021; Wang et al. 2021). Besides, lncRNA MEG3-205, NEAT1, and GAS5, have a clear opposing effect on DN by exacerbating renal fibrosis (El-Lateef et al. 2022; Feng et al. 2019; Zhou et al. 2022). In addition, lncRNAs possess a secondary structure that exhibits remarkable stability in bodily fluids, including blood and urine (Smola et al. 2016; Zhang et al. 2019). It is implied that lncRNAs possess the capability to function as biomarkers for anticipating the diagnosis and prognosis of DN, alongside serving as therapeutic drug targets.

Using lncRNA and mRNA microarrays, we found that many lncRNAs were expressed differently in plasma samples of DN. One of them was the abnormally expressed lncRNAs KIFAP3-5:1 (NONHSAT007508), which stood out and was selected for further research. The cis-regulatory prediction indicates that paired related homoeobox 1 (PRRX1) is the target gene of KIFAP3-5:1. PRRX1, a transcription factor, belongs to the paired homologous box family and is situated on chromosome 1q24. It binds to DNA through the helical structure of the homotype domain, which affects gene activation or repression (Bosada et al. 2021; Zuo et al. 2021). Previous studies have shown that PRRX1 participated in the EMT process of tumor cells, conferring tumor cell migration and invasion properties, and was a new inducer of EMT (Block et al. 2021). Further study has consistently demonstrated that the downregulation of PRRX1 enhances the re-expression of E-cadherin and cellular proliferation, while simultaneously suppressing cellular invasion and migration (Chen et al. 2021). These findings suggest that KIFAP3-5:1 may play a role in the development of EMT in DN via its interaction with PRRX1.

**Fig. 1:**
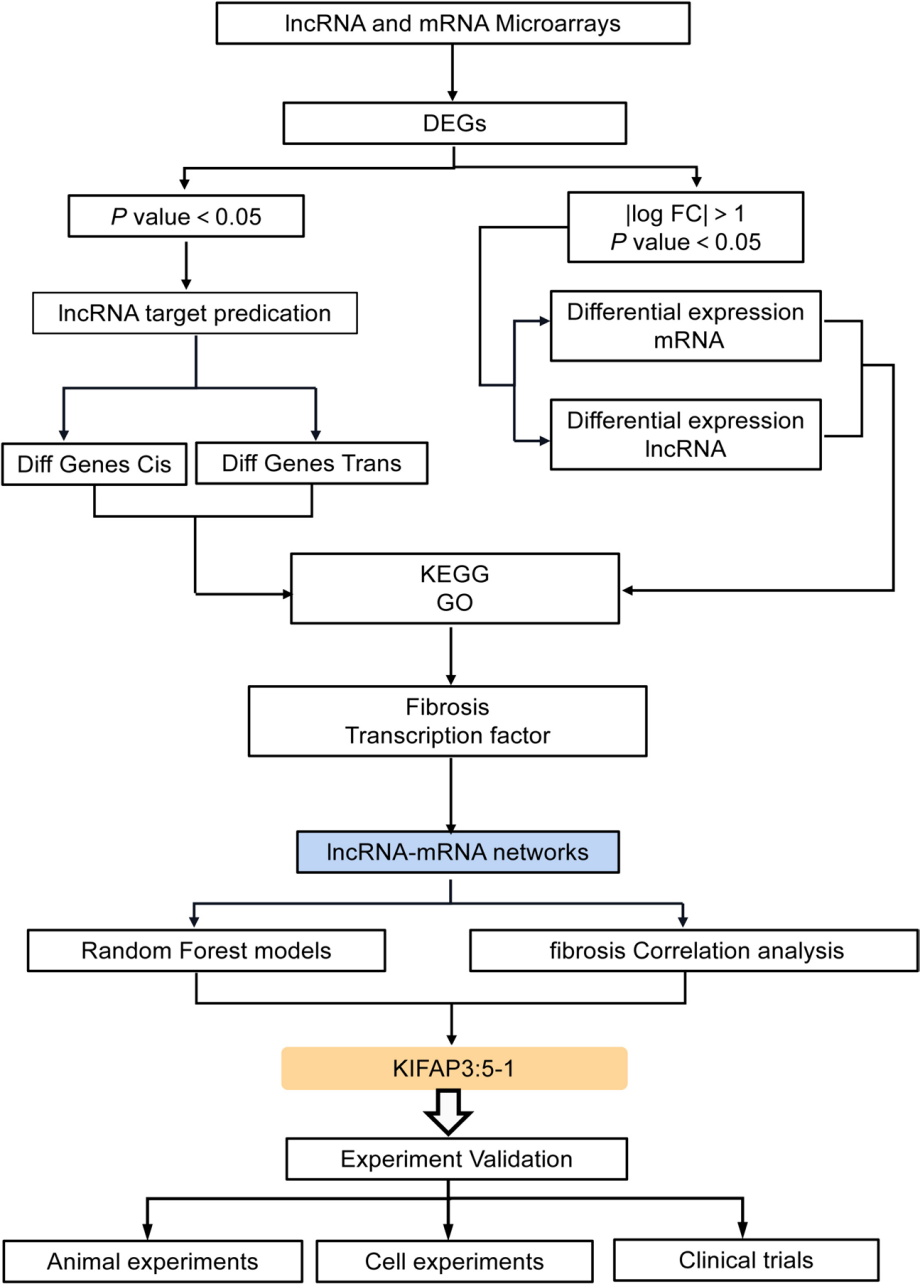
Schema of the study

In this study, we explored the function and potential molecular mechanisms of KIFAP3-5:1 in EMT and fibrosis through in vitro and in vivo experiments, and established a DN disease prediction model based on clinical patient data (Fig. 1). We hope to provide a new perspective for developing clinical treatment strategies for DN.

## Materials and methods

### Patient sample preparation

Patients who met the diagnostic criteria for type 2 diabetes mellitus (T2DM) established by the ADA were gathered from the department of endocrinology and physical examination center at the Affiliated Hospital of Xuzhou Medical University (Xuzhou, China). Thirty diabetic patients participated in this study. Fifteen of them were diagnosed with T2DM (normoalbuminuria, urine albumin creatinine ratio (UACR) less than 30 mg/g), while the remaining fifteen were identified as DN patients. Pursuant to their eGFR values, the patients were categorized into five distinct groups. Additionally, a control group consisting of 15 non-diabetic, healthy volunteers was recruited for comparative analysis.

Anticoagulated whole blood samples along with corresponding clinical information from both patients and healthy volunteers were securely retained.

### lncRNA and mRNA microarrays

Four RNA samples from15 DN patients and four RNA samples from 15 matched Ctrl subjects were selected for lncRNA and mRNA Microarray analysis. The SBC-lncRNA (human 4 × 180 k) chip provided by Shanghai Biotechnology Corporation was used to detect lncRNA and mRNA. Raw data were normalized and differentially expressed genes (DEG) were analyzed using limma (R package). Bioinformatics analysis and visualization of differential genes were performed using R package (clusterProfiler, Pheatmap and ggplot2).

### lncRNA target prediction

The cis target genes encompass those transcribed within a 10 kb window either upstream or downstream of lncRNAs, which are identified through the UCSC Genome Browser (http://genome.ucsc.edu/) and Long Target (http://www.longtarget.org). In contrast, trans target gene prediction relies on RNA duplex and mRNA sequence complementarity.

### Experimental animals

Seven-week-old C57BL/KSJ *db/db* mice with diabetes, along with their age-comparable non-diabetic littermates C57/KSJ *db/m* serving as controls, were acquired from the Nanjing Biomedical Research Institute affiliated with Nanjing University.

A comprehensive study was undertaken to assess the influence of KIFAP3-5:1 on *db/db* mice by administering lentivirus through tail vein injection. Post-injection, urine, blood, and kidney specimens were gathered over a 24-h period for subsequent analysis. The mice were injected with a precise quantity of 6 × 10^7^ TU/mouse of lentivirus via the tail vein.

### Histology analysis

A comprehensive assessment of morphological alterations in mouse kidneys was conducted utilizing PAS, HE, Masson, Sirius red staining, and immunohistochemical staining techniques. These specific methodologies have been detailed in a prior study (Liu et al. 2021).

### Immunofluorescence, IF

The previously described method of immunofluorescence staining was utilized for the staining process (Du et al. 2021). Subsequently, the stained samples were observed under an Olympus BX43F fluorescence microscope(Tokyo, Japan).

### Cell culture and treatment

Human proximal tubular epithelial cells (hRPTECs, line HK-2) were bought from American Type Culture Collection. Mouse renal tubular epithelial cells (mRTECs) were purchased from Shanghai Lianmai Biological Engineering. Cells incubated in a humidified 5% CO_2_ at 37 °C. Cultured in DMEM medium with normal glucose (5.56 mmol/L), penicillin, streptomycin, and 10% FBS. Cells passaged at 80%-90% confluence. To induce DN model, cells treated with high glucose medium for 48 h.

To investigate the role of KIFAP3-5:1 in tubular lesions, we conducted in vitro experiments using both human (hRPTECs) and mouse (mRTECs) renal tubular epithelial cells. KIFAP3-5:1 in hRPTECs and mRTECs was overexpressed or silenced by lentivirus transfection.

To definitively establish whether the impact of KIFAP3-5:1 on renal fibrosis was mediated by its interaction with PRRX1, we employed small interfering RNA to specifically silence the expression of PRRX1 in hRPTECs.

### Western blot, WB

Total proteins from renal cortex and cells were lysed and assayed using the BCA Protein Assay Kit (Thermo, USA) according to the manufacturer's instructions. Proteins were separated by SDS-PAGE and transferred to nitrocellulose membranes saturated with 1% BSA at 37 °C for 1 h. Antibodies used in the study included β-actin (CST, USA, 1:10,000), ZO-1 (CST, USA, 1:1000), E-Cadherin (Bioworld, China, 1:400), Vimentin (Affinity, China, 1:1000), α-SMA (Affinity, China, 1:1000), and PRRX-1(Abcam, USA, 1:5000). Band densities were quantified using the Odyssey instrument (Gene Corporation, USA) and normalized to β-actin internal control.

### qRT-PCR

The total RNA was isolated from plasma employing the QRIzol reagent (QIAGEN, Germany). Subsequently, 80 ng of the isolated RNA were used to synthesize cDNA through reverse transcription, utilizing the QuantiNova Reverse Transcription Kit (QIAGEN, Germany). This process was carried out in strict accordance with the manufacturer's recommended protocol.

The extraction of total RNA from both hRPTECs and mRTEC was performed using the Trizol reagent (Invitrogen, USA), strictly adhering to the manufacturer's guidelines. The qRT-PCR procedure was executed precisely as outlined in previous reports. The complete sequence of the lncRNA was retrieved from various databases, including Ensemble (Cunningham et al. 2022), LNCimedia (Volders et al. 2019), and NCBI (https://www.ncbi.nlm.nih.gov/gene/?term). The primers necessary for the study were designed and synthesized by Sangon Biotech (Shanghai, China).

Cytoplasmic and nuclear RNA were extracted from hRPTECs and mRTEC using PAKIS Kit (Thermo, USA). RNA purity and concentration were measured using Nanodrop ND-2000 (Thermo, USA). Nuclear control was U6 and cytoplasmic control was 18 s. Primer sequences are listed in Supplementary Table S3.

### Luciferase assay

The hRPTECs were transfected with a combination of a PRRX1-dependent luciferase reporter and a Renilla vector from Promega. Transfection was performed using PolyFect. Seventy-two hours later, the cells were lysed and luciferase activity was assayed using a dual-luciferase reporter assay system from Promega, following manufacturer's guidelines.

### Statistical analysis

All statistical data were expressed as mean ± standard error (mean ± SEM). Measurement data underwent testing via Student's t-test or one-way ANOVA. Enumeration data were analyzed using the chi-square test or Fisher's Exact test. Spearman correlation analysis were employed to assess the correlation between the data. The correlation analysis data of target genes and fibrosis related coding genes are from the GSE142025 (Fan et al. 2019) data set of GEO database. Diagnostic efficiency of differentially expressed lncRNAs was evaluated through ROC curve analysis using MedCalc statistical software, Version 19.7.4. Machine learning model construction and performance comparison, were conducted using R 4.2.3 and R Studio.

## Results

### Differential expression of lncRNAs related to fibrosis in DN

**Fig. 2:**
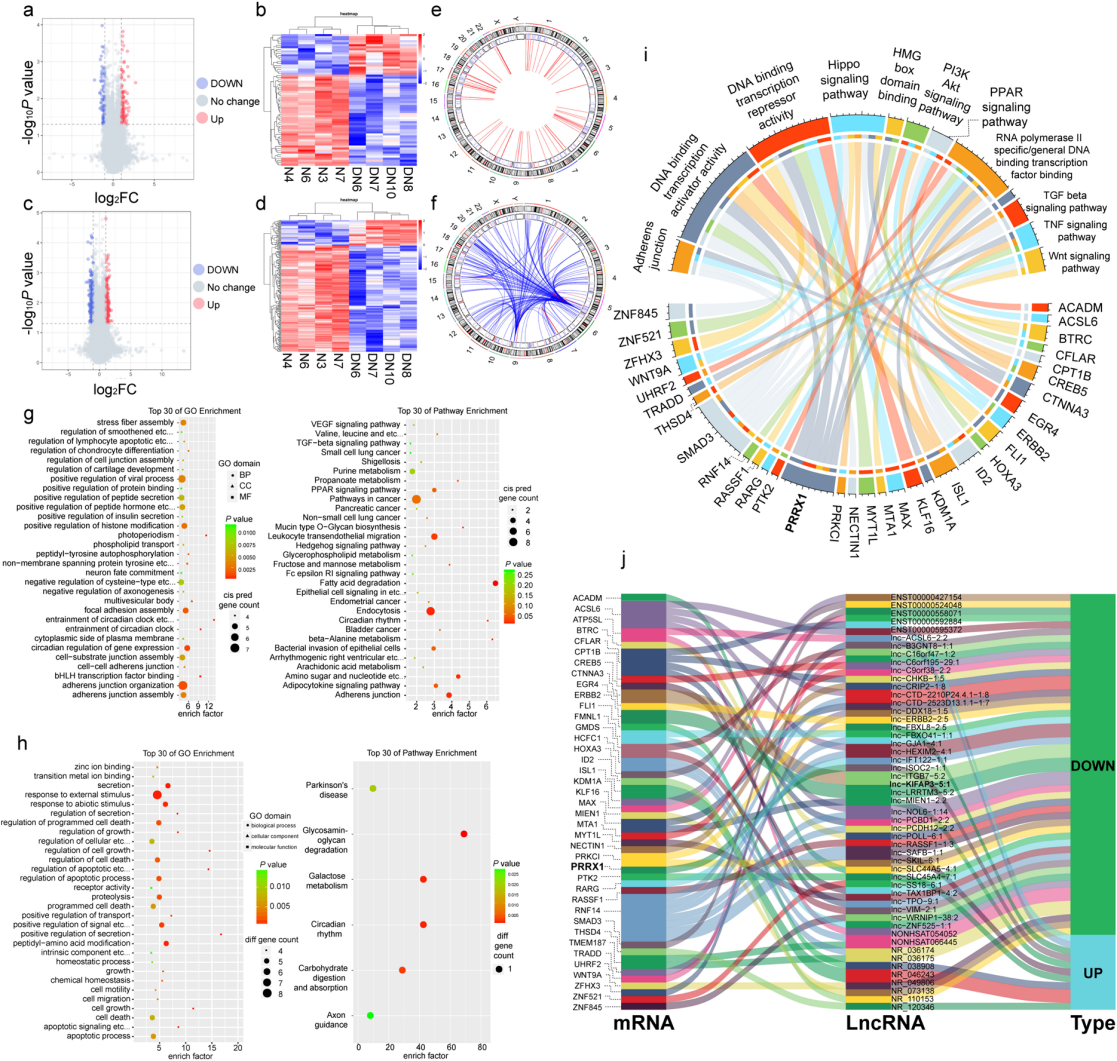
Differentially expressed lncRNAs in DN patients (**a**, **c**) Volcano plot of lncRNA and mRNA. Upregulated, red; downregulated, blue. (**b**, **d**) An expression heatmap of differentially expressed lncRNAs and mRNAs. Upregulated, red; downregulated, blue. (**e**, **f**) Differential expression of lncRNAs predicted by cis and trans mRNA. (**g**, **h**) The KEGG and GO analysis of differentially expressed lncRNA and mRNA. (**i**) The mRNAs enriched in transcriptional regulation and fibrosis related pathways. (**j**) The lncRNA corresponding to cis and trans predictions of enriched mRNA in Fig. 2 i

To identify the potential involvement of lncRNAs in DN, we performed a microarray profiling of lncRNAs and mRNAs in the plasma of DN patients and healthy, age matched controls. The basic information of DN patients and control group volunteers is shown in Table S1. Microarrays analysis revealed the presence of 77,126 lncRNAs, with 586 exhibiting significant differential expression in DN (determined by a threshold of |log_2_FC|> 1 and *P* < 0.05). Among these, 295 lncRNAs were up-regulated, while 291 were down-regulated (Fig. 2a, b, c, d). Because lncRNA lacks the ability to encode proteins, we employed lncRNA target gene prediction methods (both cis and trans) to determine its function (Fig. 2e, f). The target genes predicted by lncrna and differentially expressed genes (DEG) were enriched by KEGG and GO (Fig. 2g, h). We found that the mRNA predicted by differential lncRNA is mainly enriched in fibrosis and transcriptional regulation related items, such as Adherens junction, TGF-β signaling pathway and DNA binding transcription activator activity (Fig. 2i). We identified 51 lncRNAs related to fibrosis and transcriptional regulation through this method (Fig. 2j).

**Fig. 3:**
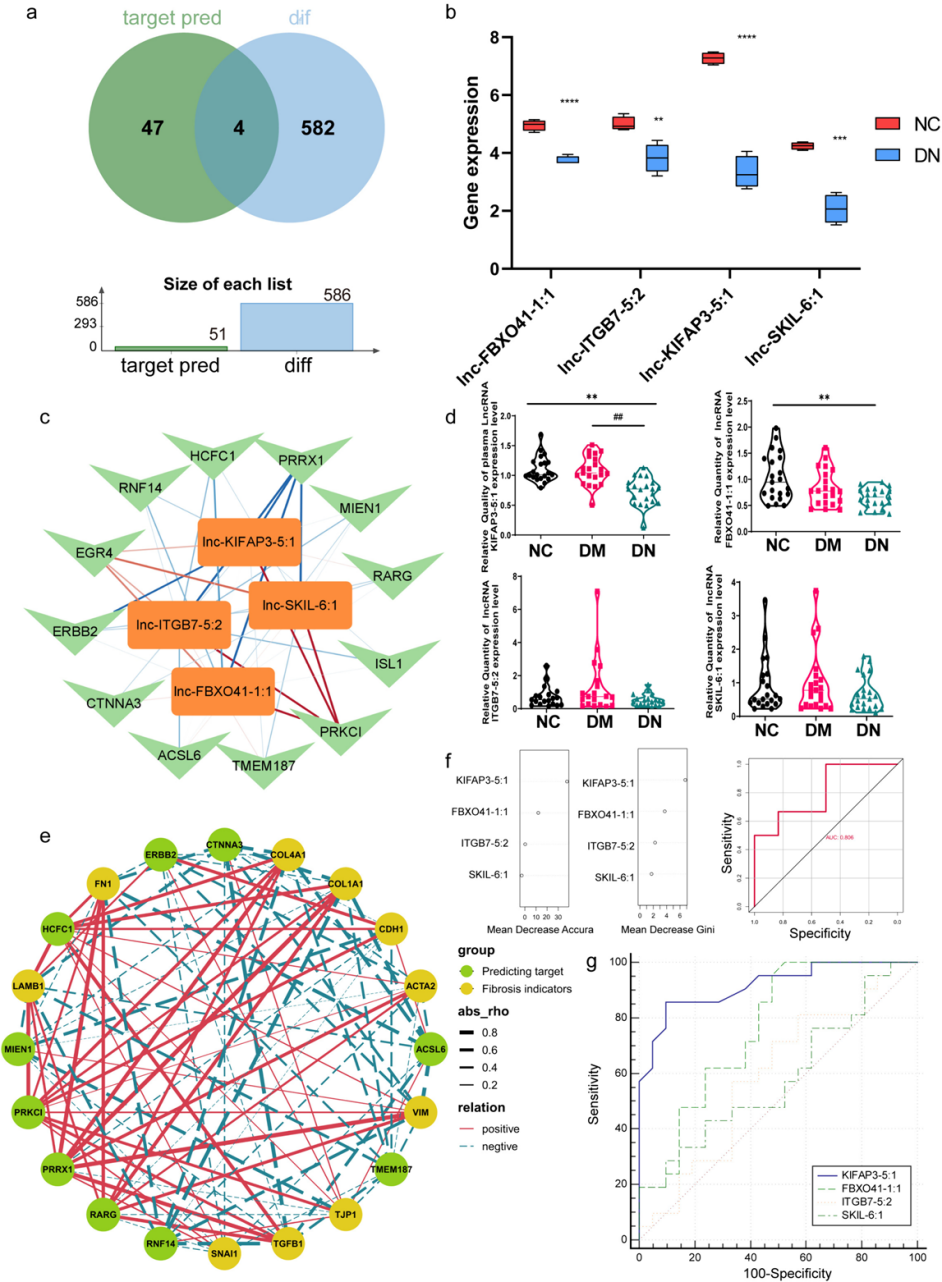
Differential expression of lncRNA related to fibrosis in DN (**a**) The Venn plot of predicted lncRNAs and differentially expressed lncRNAs related to fibrosis and transcriptional regulation. (**b**) The expression levels of 4 differentially expressed lncRNAs related to transcriptional regulation and fibrosis were determined by taking the intersection from the Venn plot in Fig. 3a. (**c**) Transcriptional regulation and fibrosis related lncRNA and mRNA correlation network. (**d**) Expression levels of four differentially expressed lncRNAs related to fibrosis and transcriptional regulation in plasma of patients with an expanded clinical cohort. (**e**) The correlation network between target genes of 4 lncRNAs and expression levels of fibrosis indicator genes. (**f**) The Variable Importance and ROC Curve of Four lncRNAs in Random Forest Model. (**g**) The ROC curve analysis of 4 lncRNAs. ^*^P < 0.05, ^**^P < 0.01, ^***^P < 0.005, ^****^P < 0.001,DN compared with NC. ^##^*P* < 0.01, DN compared with DM

Further, by intersecting 51 lncRNAs related to fibrosis and transcriptional regulation with 586 differentially expressed lncRNAs, 4 intersecting lncRNAs were obtained (Fig. 3a). Upon comparing the expression levels of lncRNAs, we observed a reduction in the expression of all four lncRNAs in the DN group compared to the normal control group. Notably, the most prominent decrease was observed in KIFAP3-5:1 (Fig. 3b). Furthermore, through expression level correlation analysis, we plotted an lncRNA mRNA correlation network diagram and found that PRRX1 was significantly negatively correlated with four lncRNAs (blue line), while PRKCI was positively correlated with four lncRNAs (red line) (Fig. 3c). However, the whole blood validation of the expanded clinical patient cohort found that the expression of KIFAP3-5:1 in the whole blood of patients with DN was significantly lower than that of the normal group and patients with diabetes without complications, while the lncRNA ITGB7-5:2 and SKIL-6:1 were not further validated (Fig. 3d). Furthermore, based on the expression levels of four lncRNAs, we determined through machine learning that KIFAP3-5:1 had the highest weight in the DN prediction model established based on the Random forest algorithm (Fig. 3f). Through ROC curve analysis, we determined that KIFAP3-5:1 had the best diagnostic performance among the four lncRNAs (Fig. 3g). After obtaining the GSE142025 (Fan et al. 2019) dataset from the GEO database, we conducted a rigorous correlation analysis between the expression levels of four lncRNA cis and trans predicted mRNA, as well as fibrosis-related coding genes. This approach allowed us to gain a deeper understanding of the interactions and relationships among these genes. Upon analysis, we discovered a notable positive correlation between PRRX1 and several coding genes, namely FN1, COL4A1, COL1A1, VIM, and TGFB1, which serve as fibrosis-related indicators (Fig. 3e).

### KIFAP3-5:1 is down-expressed in diabetic nephropathy

**Fig. 4:**
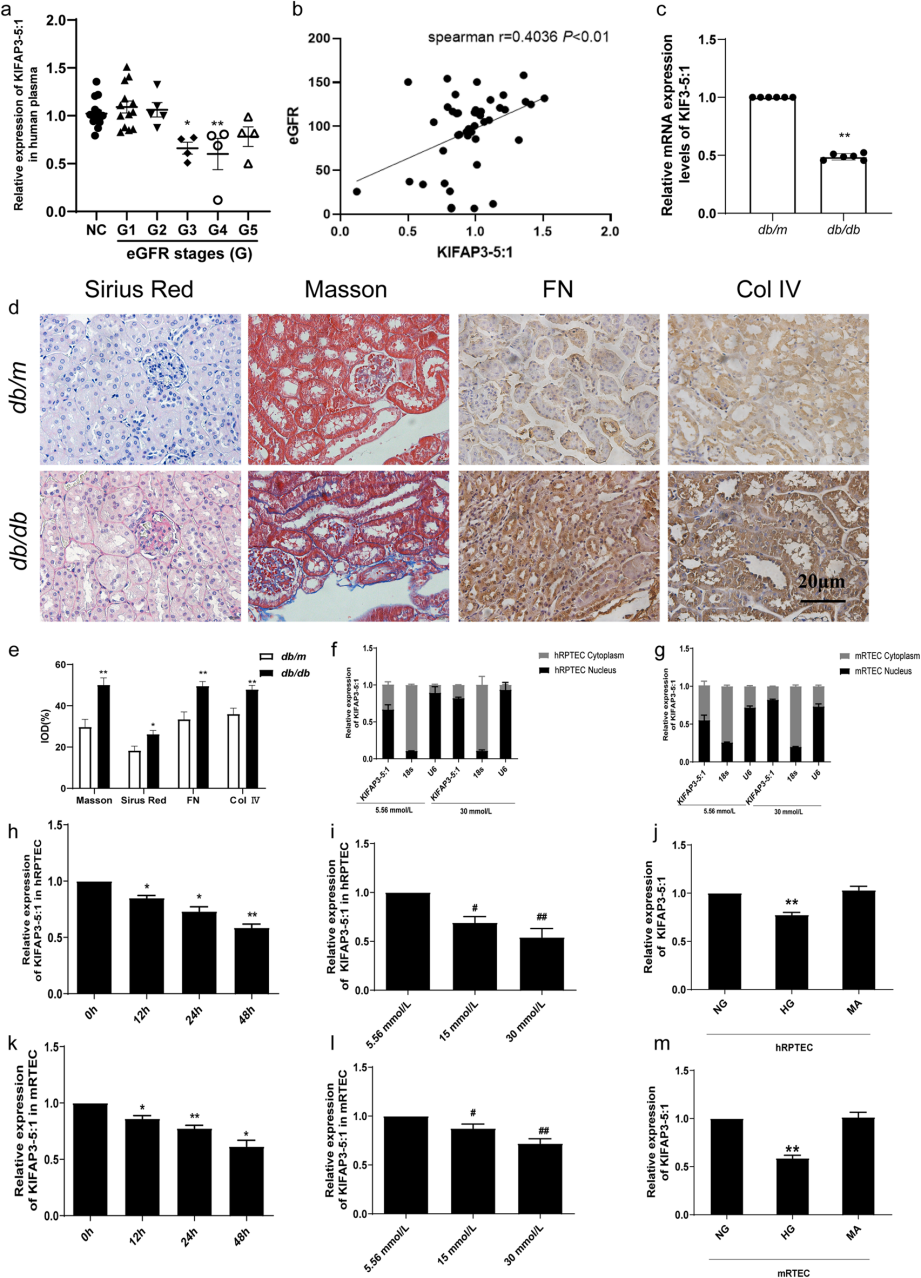
The expression and characterization of KIFAP3-5:1 in vivo and in vitro and its correlation with EMT (**a**) KIFAP3-5:1 mRNA levels in different stages of DN. Data are expressed as the mean ± SEM, n = 45, *P < 0.05, **P < 0.01 vs NC. (**b**) Linear regression shows a positive correlation between plasma KIFAP3-5:1 mRNA levels and kidney function (eGFR), n = 45. (**c**) Level of KIFAP3-5:1 mRNA expression level in *db/m* and *db/db* mice. Data are expressed as the mean ± SEM,n = 6, ^*^P < 0.05, ^**^P < 0.01 vs *db/m*. (**d**) Morphology and fibrosis in *db/db* mice. Sirius red staining, Masson, FN and Col IV of kidney tissues; shown are representative images from 6 mice per group. (**e**) Quantifications of positive region from different groups of mice. (**f**, **g**) The expression level of KIFAP3-5:1 in cytoplasm and nuclei of HG-treated hRPTECs and mRTECs, respectively. 18 s (cytoplasm retained) and U1 (nuclear retained) were used as controls. (**h**, **k**) mRNA expressions of KIFAP3-5:1 in hRPTECs and mRTEC cells exposed to 30 mmol/L glucose for (0, 12, 24, 48 h). (**i**, **l**) mRNA expression of KIFAP3-5: 1 in hRPTECs and mRTECs exposed to 5.56 mmol/L, 15 mmol/L, and 30 mmol/L glucose for 48 h. (**j**, **m**) mRNA expressions of KIFAP3-5:1 in hRPTECs and mRTECs exposed to 30 mmol/L high glucose and mannitol. Data are expressed as the mean ± SEM, n = 3. ^*^P < 0.05, ^**^P < 0.01. HG compared with NG. 12 h, 24 h, 48 h compared with 0 h. ^#^*P* < 0.05, ^##^*P* < 0.01, 15 mM,30 mM,60 mM compared with 5.56 mM. NG: normal glucose group (5.56 mM), HG: high glucose group (30 mM), MA: mannitol group (30 mM)

In clinical practice, the diagnosis of DN is determined by the persistent presence of increased albuminuria and/or a decrease in eGFR. For the purposes of our study, DN patients were categorized into five distinct groups based on their eGFR levels. After conducting a thorough analysis, we discovered a notable decrease in the plasma KIFAP3-5:1 level among patients diagnosed with stage G3 DN, in comparison to the healthy control group. Furthermore, our findings indicate that the expression level of KIFAP3-5:1 is closely linked to the clinical progression of DN (Fig. 4a). Besides, KIFAP3-5:1 levels were positively associated with eGFR (Fig. 4b). The qRT-PCR analysis revealed a substantial decrease in the expression level of KIFAP3-5:1 in the kidneys of *db/db* mice, compared to their *db/m* counterparts (Fig. 4c). The Masson and Sirius red staining revealed an elevated accumulation of collagen fibers within the renal tubules of *db/db* mice. Additionally, immunohistochemical staining demonstrated an augmented expression of FN and Col IV in the kidneys of these mice (Fig. 4d, e). These suggest that lncRNA KIFAP3-5:1 may be related to fibrosis.

We conducted a detailed analysis of the subcellular localization of KIFAP3-5:1 within renal tubular epithelial cells, under both normal glucose (NG) and high glucose (HG) conditions. The qRT-PCR analysis revealed the presence of KIFAP3-5:1 in both the nuclear and cytoplasmic compartments of renal tubular epithelial cells. However, a significantly elevated expression of KIFAP3-5:1 was observed in the nucleus compared to its expression in the cytoplasm (Fig. 4f, g). It suggested that KIFAP3-5:1 was predominantly distributed in the nucleus of renal tubular epithelial cells. To investigate the potential association between the downregulation of KIFAP3-5:1 and renal tubular cells in DN, hRPTECs were incubated with varying concentrations of glucose solution (5.56 mmol/L, 15 mmol/L, 30 mmol/L) for durations of 0 h, 12 h, 24 h, and 48 h. Exposure to high glucose concentrations led to a dose- and time-dependent reduction in KIFAP3-5:1 levels. Its expression level reached lowest point at the concentration of 30 mmol/L and the incubation time of 48 h (Fig. 4h, i). It is noteworthy that the osmotic pressure resulting from a concentration of 30 mmol/L did not exert any influence on the expression of KIFAP3-5:1 in the present study (Fig. 4j). According to these results, a 48 h stimulation with a HG concentration of 30 mmol/L was selected for the next experiments. To determine whether the alterations observed in KIFAP3-5:1 are a consistent characteristic of renal tubular epithelial cells exposed to HG, we conducted in vitro studies employing an alternative renal tubular epithelial cell line, the mRTECs. The results in mRTECs were consistent with in hRPTECs (Fig. 4k, l, m). These findings collectively indicate that KIFAP3-5:1 may serve as a DN-associated factor and play a crucial role in both EMT and the renal fibrosis process.

### KIFAP3-5:1 ameliorates EMT in renal tubular epithelial cells under high glucose conditions

**Fig. 5:**
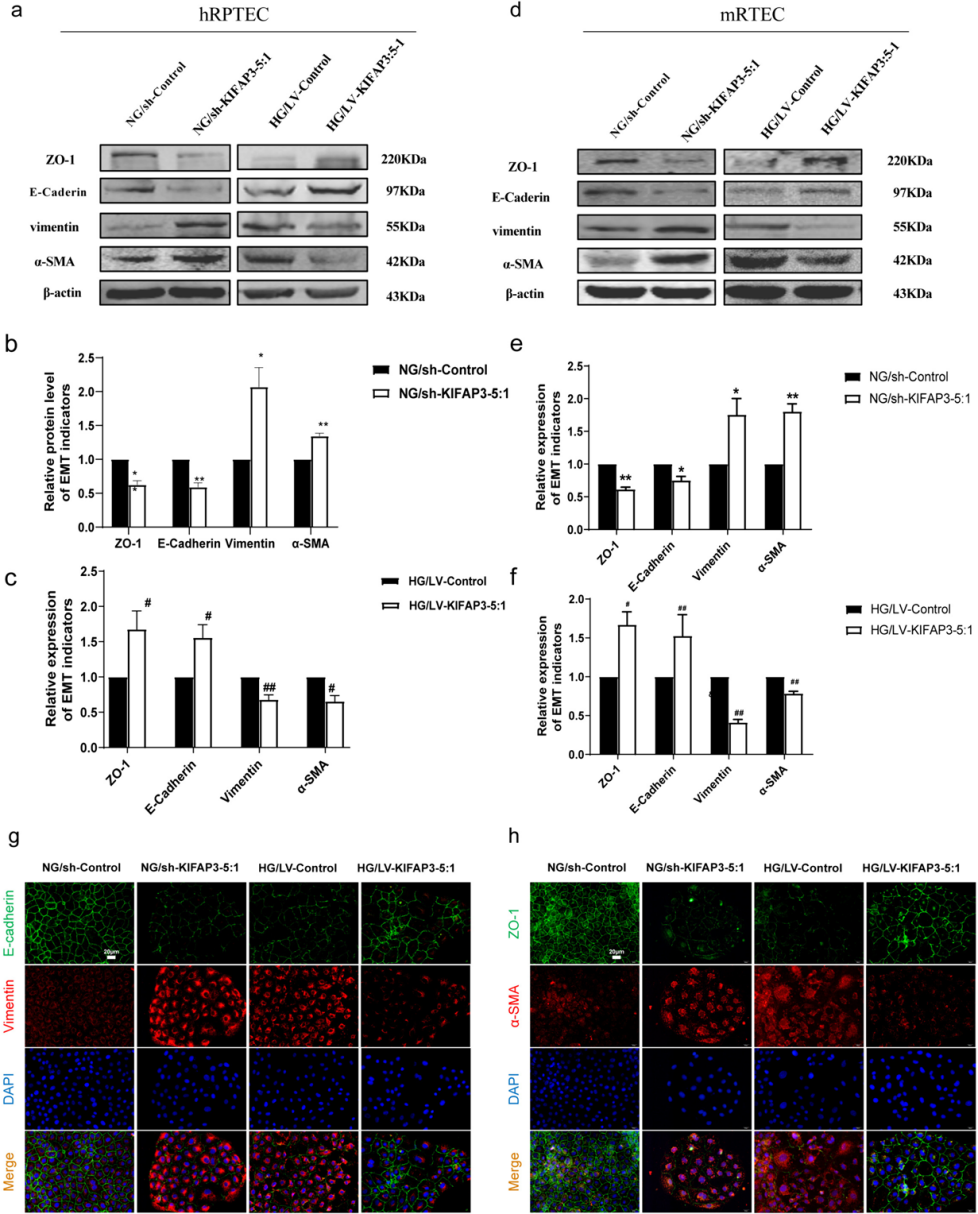
Effect of KIFAP3-5:1 on EMT induced by high glucose in human and murine renal tubular epithelial cell (**a**,**b**, **c**) The relative protein levels of EMT-associated proteins in hRPTECs. (**d**, **e**, **f**) The relative protein levels of EMT-associated proteins in mRTECs. (**g**, **h**) Distribution and expressions of E-cadherin, Vimentin, ZO-1 and α-SMA in hRPTECs cells by immunofluorescence (bar = 20 μm). Data are expressed as the mean ± SEM, n = 3. ^*^P < 0.05, ^**^P < 0.01 vs NG/sh-Control, ^#^*P* < 0.05, ^##^*P* < 0.01 compared with HG/sh-Control. NG/sh-Control: cells were transfected with the control lentivirus vector. NG/shKIFAP3-5:1: cells were transfected with KIFAP3-5:1 shRNA lentivirus vector. HG/LV-Control: cells were transfected with the control lentivirus vector. HG/LV-KIFAP3-5:1: cells were transfected with KIFAP3-5:1 overexpressed lentivirus vector

The renal tubular epithelial cells' EMT process serves as an early indicator of renal fibrosis. To elucidate the role of KIFAP3-5:1 in renal tubular epithelial cells, we initially engineered lentiviruses to modulate its expression levels. As shown in Fig. S1, overexpression of KIFAP3-5:1 was achieved in hRPTECs and mRTECs using LV-KIFAP3-5:1, and KIFAP3-5:1 was knocked down using LV-shKIFAP3-5:1. After conducting a thorough analysis of the expression patterns of key proteins involved in EMT, our findings indicated that the downregulation of KIFAP3-5:1 led to a substantial reduction in the expression of epithelial markers such as E-cadherin and ZO-1, whereas it concurrently caused a marked elevation in mesenchymal markers, including α-SMA and vimentin, when compared to the respective control group. However, the expressions of epithelial cell markers and mesenchymal markers were reversed when KIFAP3-5:1 was overexpressed in both the HG‐treated hRPTECs and mRTECs (Fig. 5a-h). In conclusion, our results revealed that KIFAP3-5:1 knocking-down promoted the EMT procession, while elevated expressions of KIFAP3-5:1 reduced renal tubular EMT caused by high glucose.

### KIFAP3-5:1 inhibits EMT of renal tubular epithelial cells by negatively regulating PRRX1

**Fig. 6:**
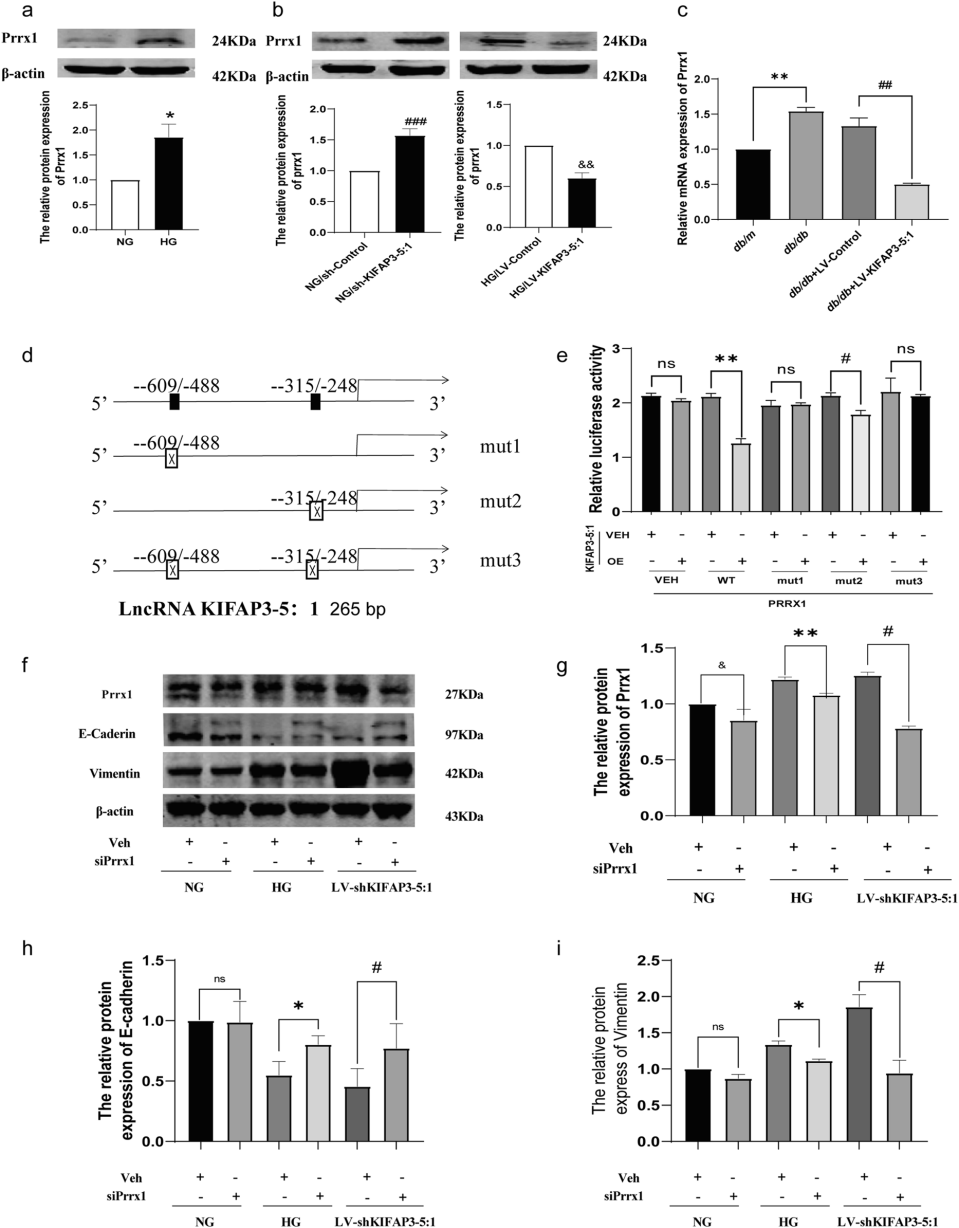
The effects of KIFAP3-5:1 on EMT of renal tubular epithelial cells through PRRX1 (**a**) The relative protein levels of PRRX1 in hRPTECs. (**b**) The protein expression of PRRX1 regulated by KIFAP3-5:1 lentivirus or its shRNA. (**c**) The mRNA expression of PRRX1 regulated by KIFAP3-5:1 lentivirus or its shRNA. (**d**) Schematic representation of luciferase construct of PRRX1 promoter and the mutation sites of − 609/ − 488 and − 315/ − 248 binding sites. (**e**) Dual-luciferase reporter assay was performed to measure the luciferase activity in hRPTECs Co-transfection of control or KIFAP3-5:1 overexpressed lentivirus and pGL3-PRRX1 with or without mutations. Data are mean ± SEM. n = 3. ^*^P < 0.05, ^**^P < 0.01 compared with wt NC group; ^#^*P* < 0.05, ^##^*P* < 0.01 compared with mut2 NC group. (f, g, h, i) The relative protein levels of EMT-related protein in hRPTECs infected with control or LV-shKIFAP3-5:1 lentiviral particles in addition to PRRX1 siRNAs or control, followed by treatment with HG. Data are expressed as the mean ± SEM, n = 3. ^&^*P* < 0.05, ^&&^ *P* < 0.01 compared with NG + siPRRX1-veh, ^*^P < 0.05, ^**^ *P* < 0.01 compared with HG + siPRRX1-veh, ^#^*P* < 0.05, ^##^*P* < 0.01 compared with LV-shKIFAP3-5:1 + siPRRX1-veh, ns: no significant difference

In order to investigate the underlying mechanism of KIFAP3-5:1 in EMT and renal fibrosis associated with DN, we employed the Long Target database (http://www.longtarget.org) to identify potential target genes of KIFAP3-5:1. Based on predictive analysis, the promoter region of PRRX1 is hypothesized to serve as a potential binding site for KIFAP3-5:1. This prediction is supported by the identification of two distinct regions within the PRRX1 promoter (-488 to -609 and -248 to -315) that exhibit sequence complementarity with KIFAP3-5:1 (Fig. S2). Furthermore, the expression level of PRRX1 demonstrated a notable elevation in hRPTECs cultured under conditions of elevated glucose, exhibiting a contrasting expression pattern relative to KIFAP3-5:1 expression in DN (Fig. 6a). We next tried to verify whether a regulatory relationship existed. The knockdown of KIFAP3-5:1 resulted in an increase in the expression of PRRX1 compared to the NG groups. Conversely, the overexpression of KIFAP3-5:1 led to a decrease in PRRX1 expression in cells cultured under high glucose conditions (Fig. 6b). Consistent with in vitro experiments, KIFAP3-5:1 significantly reduced the mRNA levels of PRRX1 in *db/db* mice (Fig. 6c). We preliminary concluded that PRRX1 was negatively regulated by KIFAP3-5:1.

To determine whether PRRX1 was directly repressed by KIFAP3-5:1, luciferase reporter gene constructs were generated for the PRRX1 core promoter fragment (− 1192 to + 1, WT) and corresponding fragments harboring mutations in the − 609/ − 488 (mut1) and − 315/ − 248 binding elements (mut2), as well as a double mutation of these elements (mut3) (Fig. 6d). The luciferase activity of PRRX1-wt and − 315/ − 248 individual mutant were significantly decreased by KIFAP3-5:1 (Fig. 6e, second and fourth set), while KIFAP3-5:1 had no influence on the − 609/ − 488 individual mutant or double mutation of these elements (Fig. 6e, third and fifth set). The findings suggest a direct interaction between PRRX1 and KIFAP3-5:1 at the − 609/ − 488 elements.

To investigate the role of PRRX1 in EMT, we further silenced PRRX1 using siRNA in hRPTECs treated with high glucose or LV shKIFAP3-5:1 (Fig. 6f, g). The findings indicate that when compared to the NG group, exposure to high glucose levels or LV shKIFAP3-5:1 results in a decrease in the protein expression of E-cadherin and a concurrent increase in the expression level of vimentin. These alterations are associated with the induction of EMT. The further suppression of PRRX1 expression under conditions of high glucose or LV shKIFAP3-5:1 has the potential to elevate the expression of E-cadherin, decrease the expression of vimentin, and postpone the progression of EMT (Fig. 6h, i). The available data indicate that KIFAP3-5:1 effectively inhibits the EMT of renal tubular epithelial cells by directly modulating the expression of PRRX1.

### KIFAP3-5:1 ameliorates renal function, EMT and renal interstitial fibrosis in *db/db* mice

**Fig. 7:**
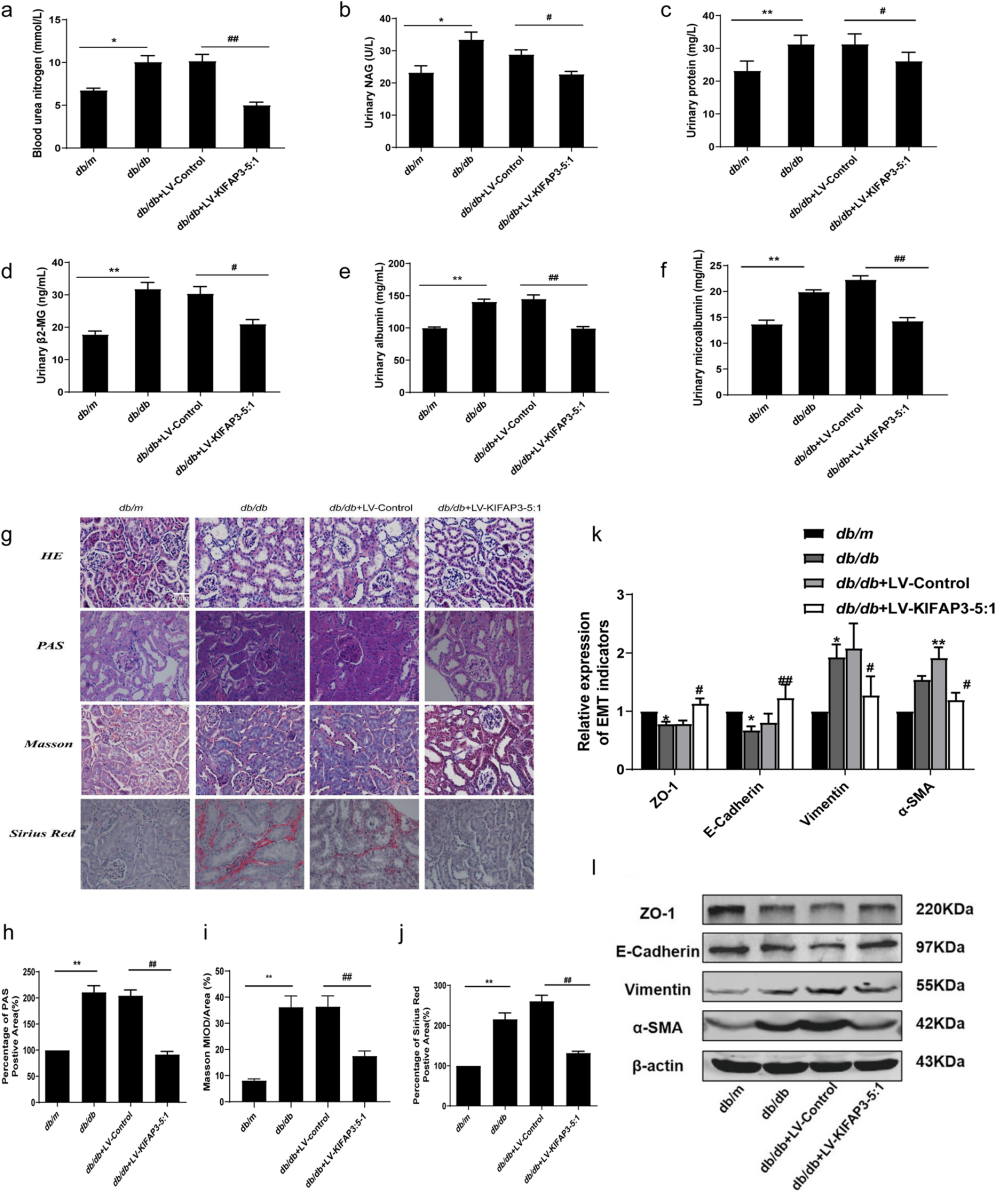
Effect of KIFAP3-5:1 on renal function and renal interstitial fibrosis in *db/db* mice (**a**, **b**, **c**, **d**, **e**, **f**) Graphic presentation shows (**a**) blood urea nitrogen (BUN) levels, (**b**) NAG, (**c**) urinary protein, (**d**) β2-MG, (**e**) urinary albumin. (**f**) urinary microalbumin in different groups as indicated. (**g**, **h**, **i**, **j**) HE, PAS (**h**), Masson (**i**) and Sirius red staining (**j**) of renal cortex sections of mice. Scale bar = 20 μm. (k, l)The relative protein levels of EMT-associated proteins in mice. Data are expressed as the mean ± SEM, n = 6. ^*^P < 0.05, ^**^P < 0.01 compared with *db/m*. ^#^*P* < 0.05, ^##^*P* < 0.01 compared with *db/db* + LV-Control. *db/m*: normal mice group, *db/db*: diabetic mice group, *db/db* + LV-Control: Lnc-KIFAP3-5:1 overexpression control group. *db/db* + LV-KIFAP3-5:1: Lnc-KIFAP3-5:1 overexpression lentivirus-injected group

Upon establishing the significance of KIFAP3-5:1 in the advancement of EMT, we proceeded to investigate its potential to improve renal function and alleviate renal interstitial fibrosis in *db/db* mice. LV-KIFAP3-5:1 was injected to increase the expression of KIFAP3-5:1 in *db/db* mice. As demonstrated in Fig. S3, a marked elevation in the mRNA expression levels of KIFAP3-5:1 was observed in the kidneys of *db/db* mice treated with LV-KIFAP3-5:1, in comparison to the control group treated with *db/db* + LV. When comparing *db/m* mice with *db/db* mice, it was observed that the levels of FBG, weight, and kidney weight index increased in the latter. Nevertheless, the overexpression of KIFAP3-5:1 did not exhibit any significant influence on the FBG and weight of *db/db* mice, but interestingly, it led to a decrease in the kidney weight index of these mice (Table S2). The renal function is primarily gauged by BUN, UP, and mAlb. Additionally, β-MG and NAG serve as reliable markers for renal tubular injury. When comparing the *db/m* mice to the *db/db* mice, it was observed that the levels of BUN, β-MG, UP, and mAlb were significantly elevated in the latter. Notably, the overexpression of lncRNA KIFAP3-5:1 resulted in an improvement in renal function levels among the *db/db* mice (Fig. 7a-f).

To gain a deeper understanding of the potential role played by KIFAP3-5:1 in renal fibrosis of diabetic nephropathy, we employed histological staining techniques such as HE, PAS, Masson, and Sirius red staining to assess the pathological alterations in the kidney tissue of mice. As shown in Fig. 7g-j, compared with *db/m* mice, the renal tissue of *db/db* mice showed mesangial hyperplasia, basement membrane thickening, deposition of glycogen and collagen fibers, and overexpression of KIFAP3-5:1 significantly improved these changes.

The effect of KIFAP3-5:1 on renal fibrosis in *db/db* mice was assessed by examining the expression of EMT marker proteins. In comparison to *db/m* mice, a notable decrease was observed in the expression of ZO-1 and E-cadherin in *db/db* mice, whereas a significant increase was noted in the expression of Vimentin and α-SMA. Introduction of overexpressed KIFAP3-5:1 in *db/db* mice resulted in an elevation of ZO-1 and E-cadherin expression, a concurrent reduction in Vimentin and α-SMA expression, and a delayed progression of EMT (Fig. 7k, l). In summary, these data suggest that KIFAP3-5:1 can improve renal function, slow down the occurrence of EMT and renal interstitial fibrosis in DN.

### Plasma KIFAP3-5:1 level was potential biomarkers of DN in humans

**Fig. 8:**
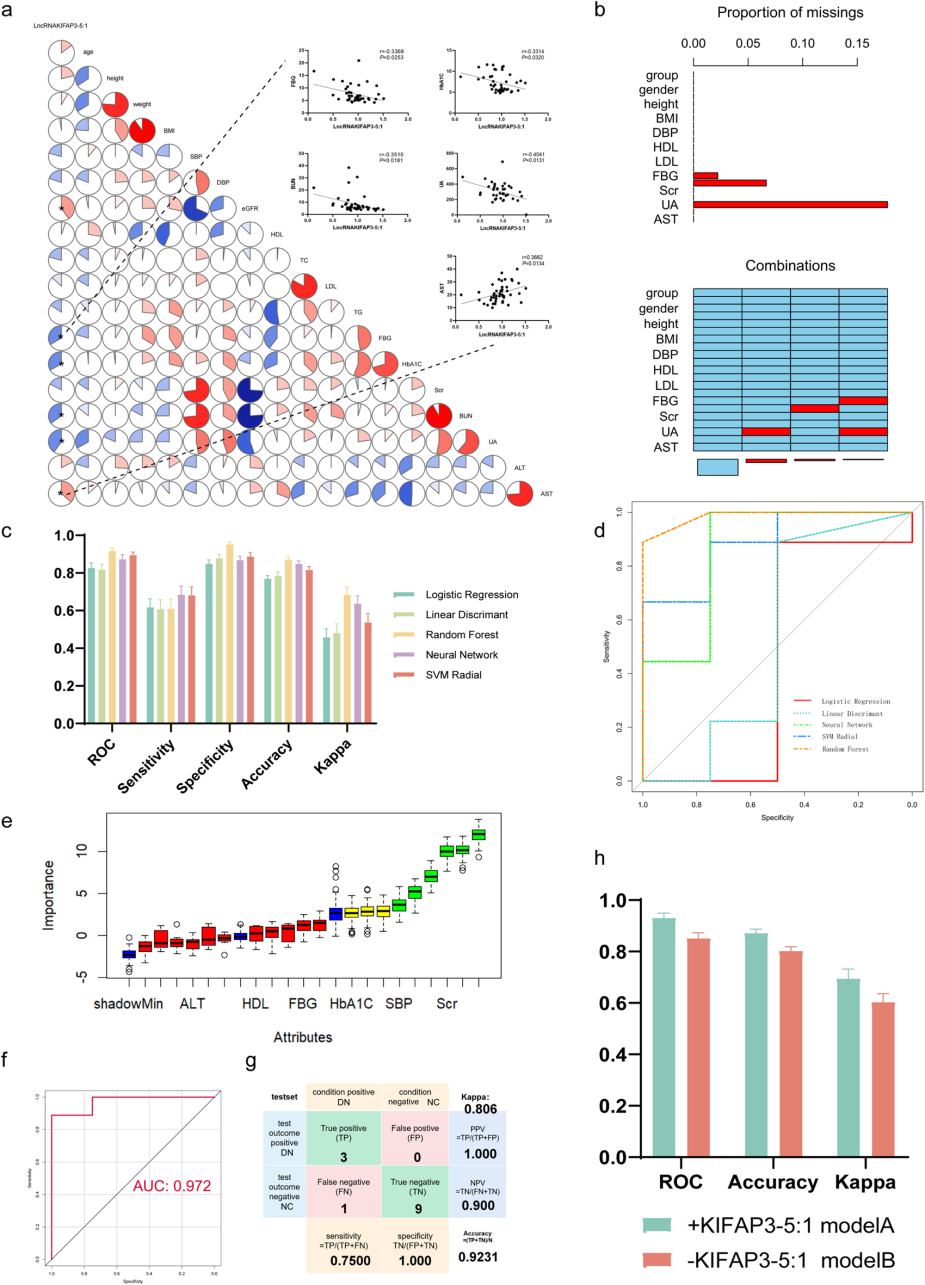
Plasma KIFAP3-5:1 is a potential new biomarker of DN in Humans (**a**) Correlation analysis between KIFAP3-5:1 and clinical indicators. (**b**) The distribution of missing values in the variables of the clinical cohort dataset. (**c**) Comparison of model performance among 5 different algorithms. (**d**) The ROC curves of 5 different algorithms. (**e**) The variable importance ranking determined by Boruta algorithm. The blue boxplot corresponds to the Z score of a shadow feature; the red and green boxplots represent the Z scores of rejected and confirmed features, respectively; and the yellow color indicates the Z scores of tentative features. (**f**) The ROC curves of the model constructed using the optimal algorithm after feature selection. (**g**) The confusion matrix of the new model constructed using the optimal algorithm after feature selection. (**h**) The comparison of performance between Model A containing KIFAP3-5:1 and Model B without the variable KIFAP3-5:1

To gain a deeper understanding of the clinical significance of KIFAP3-5:1, we proceeded to investigate the association between the expression levels of KIFAP3-5:1 in plasma and various clinical indicators. We observed a positive correlation between the plasma expression of KIFAP3-5:1 and eGFR and ALT levels. Conversely, we noted a negative association with FBG, HbA1C, BUN, and UA levels. These findings suggest a significant involvement of KIFAP3-5:1 expression in the pathogenesis of DN and renal function alterations among patients (Fig. 8a). Machine learning algorithms were further used to explore the role of KIFAP3-5:1 in the diagnosis and prediction of diabetic nephropathy. Firstly, we used the aggr function of the R Package VIM to identify missing data for clinical indicators. We found that the missing values for each indicator were below 30%, and data imputation could be performed using the R package missForest (Stekhoven and Buhlmann 2012) (Fig. 8b). Then, the R package caret was used to compare the performance of different algorithms. In the fivefold 10 repetitions, the area under the ROC curve of the model constructed by the random forest algorithm was 0.9163 ± 0.0153, the sensitivity was 0.6100 ± 0.0501, the specificity was 0.9510 ± 0.0140, and the Kappa value was 0.6833 ± 0.0412. It showed good performance among the five algorithms (Fig. 8c and d). Therefore, we ultimately decided to use the random forest algorithm to construct a DN disease prediction model. Next, we used the Boruta algorithm for feature selection. The Boruta algorithm (Kursa 2014) is a wrapper based on the random forest classification algorithm, which evaluates the importance of variables in the model by establishing random "shadow" attributes. KIFAP3.5–1, SBP, eGFR, Scr, BUN, and UA were identified as important clinical variables (Fig. 8e). We further established a new Model A using the random forest algorithm for the 6 important variables and 3 undetermined variables identified by the Boruta algorithm. As shown in Fig. 8f and Fig. 8g, using the test set to evaluate the performance of Model A, it was found that the area under the ROC curve was 0.972, the accuracy was 0.9231, and the Kappa value was 0.806. Furthermore, the DN prediction model B was established using eight other variables identified by the Boruta algorithm that did not include KIFAP3-5:1. We found that compared to Model A, Model B, which lacked KIFAP3-5:1, had lower area under ROC curve, accuracy, and Kappa values (Fig. 8h). This indicates that plasma KIFAP3.5–1 is a potential biomarker for DN, playing a crucial role in predicting the disease.

## Discussion

Diabetic nephropathy (DN), a common complication of diabetes, poses a significant threat to human health globally. Despite this, effective treatment strategies remain unavailable (Zheng et al. 2020). In the present investigation, we have identified KIFAP3-5:1 as a crucial long non-coding RNA (LncRNA) in the context of DN. Notably, the expression levels of KIFAP3-5:1 were observed to be significantly reduced in the plasma samples of DN patients. The downregulation of this expression is not only linked to the progression of proteinuria and renal dysfunction, but also holds significant diagnostic potential for patients with DN. Our findings, utilizing both in vivo and in vitro techniques, demonstrate that the overexpression of KIFAP3-5:1 effectively mitigates high glucose-induced EMT and renal fibrosis in *db/db* mice. Mechanistically, KIFAP3-5:1 exerts its beneficial effects by negatively regulating PRRX1, thereby mitigating EMT in renal tubular epithelial cells. And clinical research results indicate that plasma KIFAP3-5:1 is associated with renal function in DN patients and can serve as a disease marker to predict the occurrence of DN and assist in clinical diagnosis.

LncRNAs have been identified as crucial modulators of physiological and pathological processes in human health and disease states. Recent studies have indicated that lncRNAs play a significant role in the progression of DN. Specifically, under conditions of high glucose, the expression of lncRNA Rpph1 is upregulated in MCs, leading to the augmentation of inflammatory responses and cellular proliferation through the Gal-3/Mek/Erk signaling pathway (Zhang et al. 2019). Moreover, LncRNA 1700020I14Rik mitigates cell proliferation and fibrosis in DN through the miR-34a-5p/Sirt1/HIF-1α signaling pathway (Li et al. 2018). Currently, there is a significant gap in our understanding of the expression profile and functions of lncRNAs in kidney disease. In this study, on the basis of our lncRNA microarray data, 586 lncRNAs were differentially expressed in plasma of DN patients. In addition, the expression of 4 lncRNAs showed significant differences and were selected as candidate lncRNAs for fibrosis and transcriptional regulation. The qRT-PCR analysis revealed a consistent expression pattern among the two lncRNAs in the validation set patient cohort, with KIFAP3-5:1 exhibiting the most pronounced reduction. Interestingly, through bioinformatics methods for prediction, we found a potential binding site between lncRNA KIFAP3-5:1 and the EMT related gene PRRX1. PRRX1, as a target gene of KIFAP3-5:1, has been documented to facilitate EMT via the Wnt/β-catenin pathway in gastric cancer (Guo et al. 2015). Additionally, our analysis revealed a noteworthy direct proportionality between PRRX1 and fibrosis-related indicators encoded by FN1, COL4A1, COL1A1, VIM, and TGFB1. Consequently, we hypothesize that KIFAP3-5:1 may also play a role in EMT and serve as a crucial component in DN.

KIFAP3-5:1, a lncRNA, is located on chromosome 1. However, there is no report about KIFAP3-5:1 in DN up to now. In this study, we found that the target genes of KIFAP3-5:1 were associated with EMT. The association between EMT and the progression of renal fibrosis in DN is well-documented and widely recognized (Liu et al. 2019). Studying the role of KIFAP3-5:1 in DN via EMT, we found that knocking down KIFAP3-5:1 promoted EMT in renal tubular cells under normal glucose, while overexpressing it inhibited high glucose-induced EMT. In vivo, *db/db* mice showed decreased E-cadherin and increased α-SMA in renal cortex. Overexpression of KIFAP3-5:1 reversed these changes, improving kidney function and reducing glycogen and collagen deposition in renal cortex. In summary, KIFAP3-5:1 inhibits renal tubular EMT, improving renal function and fibrosis in *db/db* mice.

Previous research has established the role of lncRNAs and their potential mechanisms, primarily determined by their subcellular localization (Li et al. 2018; Martianov et al. 2007). The general mechanism exhibited by lncRNAs, particularly those located in the cytoplasm, involves a ceRNA mode that facilitates binding with miRNAs. On the other hand, lncRNAs present in the nucleus can bind with nuclear transcription factors (Wang et al. 2020). According to our data, KIFAP3-5:1 is primarily a nuclear lncRNA present in renal tubular epithelial cells, suggesting that its biological functions are primarily exerted through the modulation of gene transcription. Our research further revealed that KIFAP3-5:1 interacts with the nuclear transcription factor PRRX1 and can negatively regulate its expression levels. Many studies have shown that PRRX1 was a inducer of EMT in cancer (Meng et al. 2022; Ocana et al. 2012). Consistent with the aforementioned reports, our findings indicate that PRRX1 has the capacity to expedite the emergence of EMT. Additionally, to elucidate the interplay between KIFAP3-5:1 and PRRX1, we conducted a rescue experiment. These results indicate that under high glucose conditions, KIFAP3-5:1 downregulation can lead to the EMT process, but silencing the expression of PRRX1 can delay the progression of EMT. Therefore, we speculate that KIFAP3-5:1 may inhibit EMT in renal tubular epithelial cells by inhibiting the expression of PRRX1 in the nucleus.

In the pathological development of DN, a significant number of lncRNAs with significant clinical relevance have been discovered. These lncRNAs, including Malat1, ANRIL, and PANDAR, can serve as reliable markers for prognostic prediction and early diagnosis of DN (Zhao et al. 2020; Zhou et al. 2020; Zhu et al. 2022). In this study, the expression of KIFAP3-5:1 in plasma was significantly down-regulated in stage G3 of DN patients than those in healthy controls. In this stage, renal fibrosis is still in the inflammatory response phase. After active treatment, renal function can be reversed to normal. So it can be said that G3 diabetic nephropathy is a turning point of nephropathy (Stevens and Levin 2013). Although the plasma KIFAP3-5:1 levels cannot be used as an early indicator of very early stages of DN. The concentration of plasma KIFAP3-5:1 exhibited sensitivity as a marker for distinguishing early-stage from mid-to-severe stage diabetic nephropathy among patients with diabetes. This marker has the potential to identify those patients who are susceptible to microvascular complications prior to the onset of irreversible organic damage due to renal fibrosis. We found no statistically significant difference in plasma KIFAP3-5:1 levels between the normal controls and the G1\G2 group. This finding could potentially be attributed to the limited number of patients included in these groups. To further elucidate this observation, additional studies with larger sample sizes are necessary. Simultaneously, KIFAP3-5:1 expression inversely correlated with Scr, BUN and UA, but positively with eGFR in DN patients. These clinical parameters evaluated kidney function in DN. ROC analysis showed that plasma KIFAP3-5:1 levels are a useful biomarker for DN diagnosis, not necessarily in early stages. Combining KIFAP3-5:1 with traditional markers like eGFR and Scr created a superior predictive model compared to single indicator.

When interpreting these findings, it is imperative to acknowledge several limitations. Firstly, like any observational study, these results only show correlation, but do not impose any additional treatment on clinical patients. Additionally, the existence of a relatively small sample size poses another constraint. Thirdly, the levels of plasma KIFAP3-5:1 lack organ specificity, indicating potential EMT and fibrosis across multiple organs, not exclusively limited to the kidneys. Therefore, in order to accurately assess kidney damage, measurements of KIFAP3-5:1 should be evaluated alongside other markers of renal injury, including albuminuria. Furthermore, while the Microarrays data was derived from the plasma of patients, our subsequent validation was conducted on renal tubular epithelial cells. Currently, the exact connection between lower lncRNA levels in patient plasma and its reduced expression in hRPTECs is unclear. In the future, we will undertake further research endeavors aimed at elucidating the underlying mechanisms involved. In summary, the present study demonstrates that KIFAP3-5:1 represents a novel lncRNA associated with DN. This lncRNA has the capability to attenuate EMT in renal tubular epithelial cells and renal fibrosis in DN, achieved through its negative regulation of PRRX1. In addition, our research revealed that plasma KIFAP3-5:1 exhibits remarkable sensitivity and specificity in diagnosing DN, and its prediction model established in combination with other DN related clinical indicators has a good predictive effect on diabetic nephropathy. These revealed that KIFAP3-5:1 might serve as an predictive indicator and therapeutic target for DN.

## Supplementary Information

Below is the link to the electronic supplementary material.

Supplementary file1 (DOCX 1200 KB)

## Data Availability

The analyzed data sets generated during the study are available from the corresponding author on reasonable request.
