# Supplementary material for: LncRNA KIFAP3-5:1 inhibits epithelial-mesenchymal transition of renal tubular cell through PRRX1 in diabetic nephropathy
Source: Cell Biol Toxicol. 2024 Jun 13;40(1):47. doi: 10.1007/s10565-024-09874-5 (PMC11176233; doi:10.1007/s10565-024-09874-5)
Supplement: Supplementary file 1 — Supplementary file1 (DOCX 1200 KB) [file 10565_2024_9874_MOESM1_ESM.docx]

**Supplemental Figures**


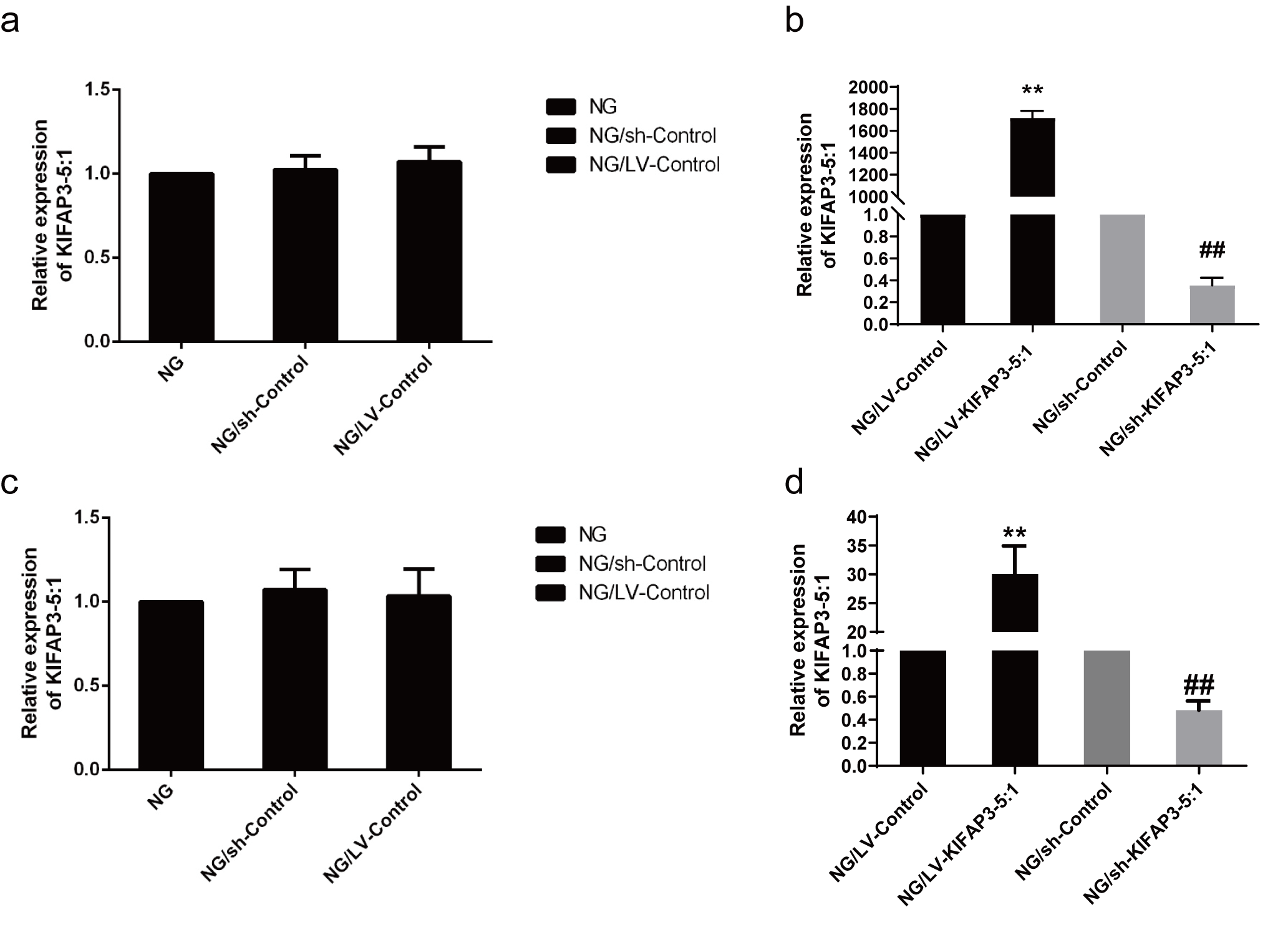


**Fig.S1 Construction of KIFAP3-5:1 overexpression and knockdown stable expression strains** (a, b)The expression of KIFAP3:5-1 was measured by qRT-PCR in hRPTECs transfected with LV-KIFAP3:5-1 or sh-KIFAP3:5-1 or their matched controls, which showed obviously upregulating or downregulating the expression of KIFAP3:5-1 compared with their matched controls, respectively. (c, d)The expression of KIFAP3:5-1 was measured by qRT-PCR in mRTECs transfected with LV-KIFAP3:5-1 or sh-KIFAP3:5-1 or their matched controls, which showed obviously upregulating or downregulating the expression of KIFAP3:5-1 compared with their matched controls, respectively. Data are expressed as the Mean±SEM, n = 3. ^*^*P* < 0.05, ^**^*P* < 0.01 vs.HG/LV-Control, ^#^*P* < 0.05, ^##^*P* < 0.01 vs.NG/sh-Control.


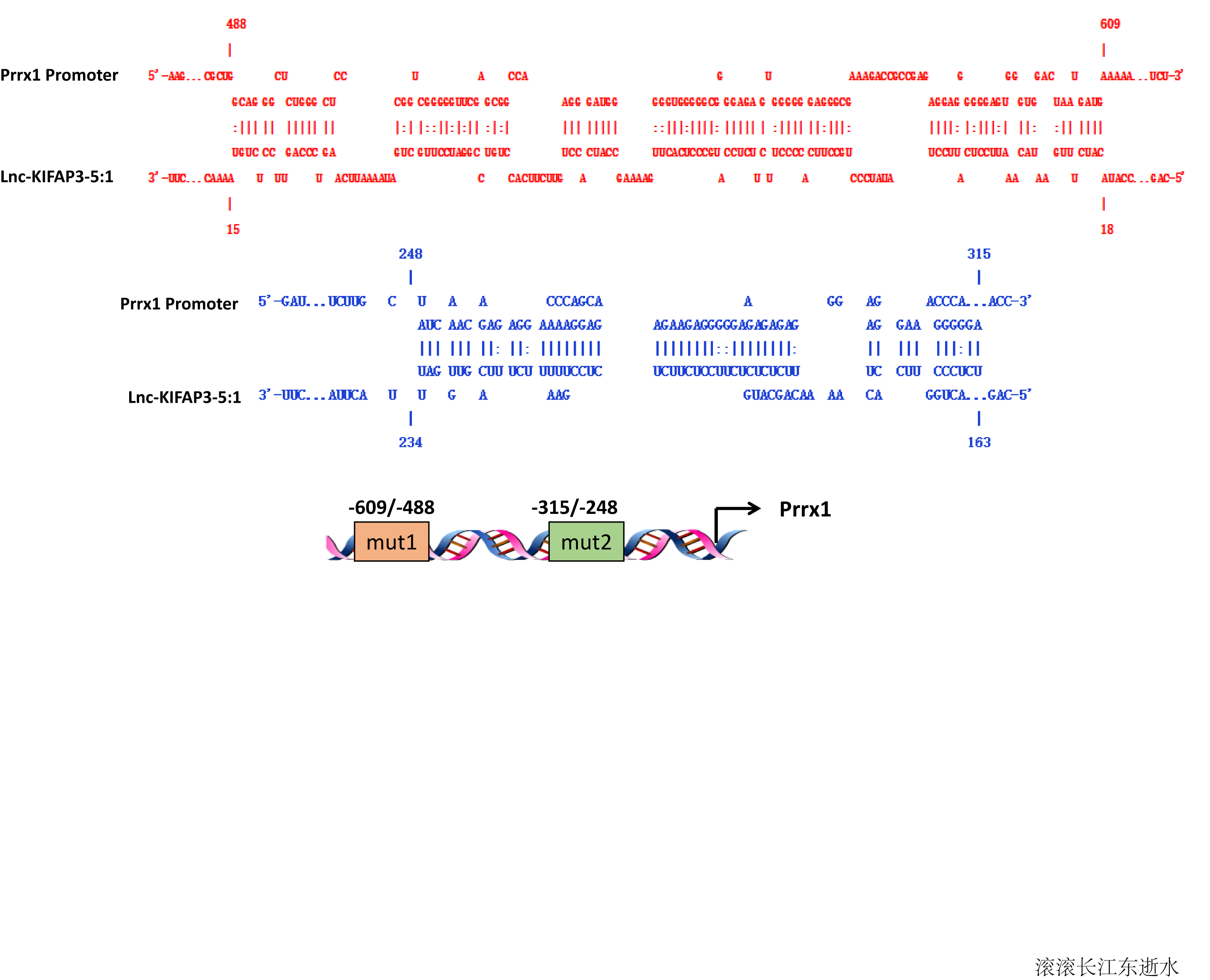


**Fig.S2 The promoter region of PRRX1 is predicted to be the binding site of KIFAP3-5:1**


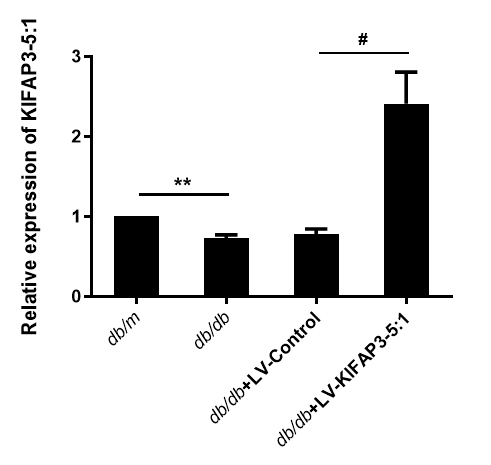


**Fig.S3 The mRNA expressions of lncRNA-KIFAP3-5:1 in the renal cortex sections of *db/db*+LV-KIFAP3-5:1 mice** Data are presented as Mean ± SEM, n=6. ^**^*P*<0.01, compared to *db/m,*^#^*P*<0.05, compared to *db/db*+LV Control.

**Table S1 The basic information of microarray participants**

| **Items** | **DN group** | **N group** | ***P*-value** |
| --- | --- | --- | --- |
| Number | 4 | 4 | - |
| Gender(male/female) | 2/2 | 2/2 | 1 |
| Age(Mean ±SEM) | 53.25 ±2.29 | 50.00 ±3.58 | 0.473 |
| HbA1c(Mean ±SEM) | 7.63 ±0.61 | 5.55 ±0.10 | 0.016* |
| CREA(Mean ±SEM) | 330.50 ±66.33 | 65.00 ±1.73 | 0.03* |

Notes: DN, diabetic nephropathy; N, healthy controls;HbA1c, Hemoglobin A1c; CREA, Creatinine. ^*^*P*<0.05, compared with N.

**Table S2 Effect of overexpression of KIFAP3-5:1 on the FBG, body weight and kidney index levels of diabetic mice**

| **Group (n=6)** | **Weight (g)** | **Blood glucose (mM)** | **Kidney Weight Index (mg/g)** |
| --- | --- | --- | --- |
| *db/m* | 25.8±0.95 | 7.57±0.32 | 11.28±0.32 |
| *db/db* | 57.5±2.06** | 29.02±0.73** | 13.17±0.20** |
| *db/db*+LV-Control | 60.78±1.82 | 28.42±1.78** | 13.24±0.50 |
| *db/db*+LV-KIFAP3-5:1 | 55.7±2.03 | 25.9±1.50 | 12.12±0.22 |

Notes: Data are expressed as the mean ± SEM, n = 6. **P* < 0.05, ^**^*P* < 0.01 compared with *db/m*. ^#^*P* < 0.05, ^##^*P* < 0.01 compared with *db/db+*LV-Control.

**Table S3 primer sequences for qRT-PCR**

| Gene |  | Primer sequence (5’to3’) |
| --- | --- | --- |
| *β-actin*  *(Homo )* | Forward: | GCAAAGACCTGTACGCCAAC |
|  | Reverse: | AGTACTTGCGCTCAGGAGGA |
| *lnc-KIFAP3-5:1*  *(Homo )* | Forward: | CAGGCCCATTTTGCCATACATCTT |
|  | Reverse: | CAGCAAGGATCCGGACAGGTGA |
| *lnc-FBXO41-1:1*  *(Homo )* | Forward: | TGTTCCAGCCATGGTGTCTC |
|  | Reverse: | GCACCAAAGTTGAGCCAGTG |
| *lnc-ITGB7-5:2*  *(Homo )* | Forward: | TGGCCACAGACAACTCAACT |
|  | Reverse: | CCTGCAGAGACTGGCTTCTAC |
| *lnc-SKIL-6:1*  *(Homo )* | Forward: | GGCTGAGGCAGGAGAATTGT |
|  | Reverse: | GGAGTCTCGCTCTTTCACCC |
| *lnc-KIFAP3-5:1*  *(Mus )* | Forward: | CCCCCTCCCTCCTCTTCTG |
|  | Reverse: | TGCAACAATCCAAGAAAATCAGT |
| *β-actin*  *(Mus )* | Forward: | AGAGGGAAATCGTGCGTGAC |
|  | Reverse: | CAATAGTGATGACCTGGCCGT |
